# The Virtual Inclusive Digital Health Intervention Design to Promote Health Equity (iDesign) Framework for Atrial Fibrillation: Co-design and Development Study

**DOI:** 10.2196/38048

**Published:** 2022-10-31

**Authors:** Nino Isakadze, Nancy Molello, Zane MacFarlane, Yumin Gao, Erin M Spaulding, Yvonne Commodore Mensah, Francoise A Marvel, Shireen Khoury, Joseph E Marine, Erin D Michos, David Spragg, Ronald D Berger, Hugh Calkins, Lisa A Cooper, Seth S Martin

**Affiliations:** 1 Division of Cardiology Department of Medicine Johns Hopkins University School of Medicine Baltimore, MD United States; 2 Ciccarone Center for the Prevention of Cardiovascular Disease Division of Cardiology, Department of Medicine Johns Hopkins University School of Medicine Baltimore, MD United States; 3 Center for Health Equity Johns Hopkins University Baltimore, MD United States; 4 School of Public Health Johns Hopkins University Baltimore, MD United States; 5 School of Nursing Johns Hopkins University Baltimore, MD United States; 6 Welch Center for Prevention, Epidemiology, and Clinical Research Johns Hopkins University Baltimore, MD United States; 7 Department of Medicine, Case Western Reserve University School of Medicine Cleveland, OH United States; 8 School of Medicine Johns Hopkins University Baltimore, MD United States

**Keywords:** atrial fibrillation, digital health intervention, human-centered design, health equity, smartphone, mobile application, cardiac, cardiology, virtual meeting, virtual health, medication adherence, health equity

## Abstract

**Background:**

Smartphone ownership and mobile app use are steadily increasing in individuals of diverse racial and ethnic backgrounds living in the United States. Growing adoption of technology creates a perfect opportunity for digital health interventions to increase access to health care. To successfully implement digital health interventions and engage users, intervention development should be guided by user input, which is best achieved by the process of co-design. Digital health interventions co-designed with the active engagement of users have the potential to increase the uptake of guideline recommendations, which can reduce morbidity and mortality and advance health equity.

**Objective:**

We aimed to co-design a digital health intervention for patients with atrial fibrillation, the most common cardiac arrhythmia, with patient, caregiver, and clinician feedback and to describe our approach to human-centered design for building digital health interventions.

**Methods:**

We conducted virtual meetings with patients with atrial fibrillation (n=8), their caregivers, and clinicians (n=8). We used the following 7 steps in our co-design process: step 1, a virtual meeting focused on defining challenges and empathizing with problems that are faced in daily life by individuals with atrial fibrillation and clinicians; step 2, a virtual meeting focused on ideation and brainstorming the top challenges identified during the first meeting; step 3, individualized onboarding of patients with an existing minimally viable version of the atrial fibrillation app; step 4, virtual prototyping of the top 3 ideas generated during ideation; step 5, further ranking by the study investigators and engineers of the ideas that were generated during ideation but were not chosen as top-3 solutions to be prototyped in step 4; step 6, ongoing engineering work to incorporate top-priority features in the app; and step 7, obtaining further feedback from patients and testing the atrial fibrillation digital health intervention in a pilot clinical study.

**Results:**

The top challenges identified by patients and caregivers included addressing risk factor modification, medication adherence, and guidance during atrial fibrillation episodes. Challenges identified by clinicians were complementary and included patient education, addressing modifiable atrial fibrillation risk factors, and remote atrial fibrillation episode management. Patients brainstormed more than 30 ideas to address the top challenges, and the clinicians generated more than 20 ideas. Ranking of the ideas informed several novel or modified features aligned with the Theory of Health Behavior Change, features that were geared toward risk factor modification; patient education; rhythm, symptom, and trigger correlation for remote atrial fibrillation management; and social support.

**Conclusions:**

We co-designed an atrial fibrillation digital health intervention in partnership with patients, caregivers, and clinicians by virtually engaging in collaborative creation through the design process. We summarize our experience and describe a flexible approach to human-centered design for digital health intervention development that can guide innovative clinical investigators.

## Introduction

### Background

Digital health interventions (DHIs) are rapidly reshaping health care, including the delivery of cardiovascular care. Health and fitness smartphone app utilization is increasing, with 84 million users overall in the United States in 2021 [1]. Racial and ethnic minorities have higher rates of smartphone use for health-related purposes than White individuals in the United States [2,3]. This represents a unique opportunity to reach diverse groups of patients outside hospital walls and empower them with disease management knowledge and tools. By engaging patients in self-management, DHIs have the potential to improve access, management, and ultimately health outcomes of patients with cardiovascular disease. There are more than 50,000 health-management apps available in the Apple and Android Google Play app stores [4,5], but the majority do not adequately engage patients [6]. This problem is likely multifactorial, with causes that include a lack of evaluation of patient needs and development of products without grounding within established behavior change techniques. To design successful DHIs, innovators would benefit from identifying challenges related to management of the disease from patient, caregiver, and clinician perspectives in the early phases of app development. Furthermore, to ensure that the end products are useful to patients across different age, race, ethnicity, sex, and socioeconomic groups, engaging diverse individuals in the design process is crucial. This approach has been supported by a randomized study in which app selection based on target users’ needs led to better usability and user satisfaction [7].

Human-centered design (HCD) is a core creative design methodology developed in the 1970s to facilitate innovative solution development processes. HCD methodology has been widely adopted in engineering and social sciences. More recently, HCD has been adopted for health care intervention development (eg, web-based health app co-design for refugee and migrant women, diabetes management app development, heart failure digital tool development, and personalized integrated care platform development for people with neurodegenerative disorders) [8-12]. To date, more than 100 design methodologies have been described that use different approaches to HCD, including participatory design and affinity diagramming, among other methodologies [9]. While there are differences in how various design methodologies are structured, many share the integral components of understanding end user needs through user engagement in the design process. DHIs developed with an HCD methodology have the potential to promote person-centered care, as they are designed in a way that respects patient needs, values, and preferences; allows care continuity outside the walls of the hospital; empowers patients with education; and provides emotional support. Overall, while general field guides for HCD are widely available, structured frameworks to guide innovators and investigators in designing disease management DHIs by applying HCD principles, including relevant stakeholders, and addressing the challenges in diverse patient engagement are limited. This provides an opportunity to describe our approach to applying HCD principles in the health care setting and contribute to the growing literature [13-15].

### Case Study Significance

Atrial fibrillation (AF) is the most common cardiac arrhythmia. The lifetime risk of AF is 1 in 5 among Black individuals and 1 in 3 among White individuals [16,17]. AF is associated with poor quality of life [18], poor subjective health [18-20], and increased morbidity and mortality [21,22]. Scientific breakthroughs in therapy (including anticoagulation and rhythm control) and interventions (including advanced ablation techniques and left atrial appendage closure) have not been uniformly adopted in practice, especially among individuals from disenfranchised racial and ethnic groups [23-28]. In order for scientific advances to reach diverse groups of patients, there is a need to identify patient-, caregiver-, and clinician-level challenges and to engage all stakeholders in the process of creating innovative solutions. We previously developed a minimally viable AF DHI by tailoring the Corrie Health digital platform. This platform consists of the patient-facing Corrie app, connected devices, and a clinician dashboard, where actionable insights are stored to inform clinical decision-making. The Corrie Health DHI was shown to successfully improve outcomes among patients recovering from myocardial infarction [29-31]. Several features of the original Corrie app were based on behavioral change theories, such as social cognitive theory and the health belief model, which includes domains of perceived susceptibility, perceived benefits, barriers, cues to action, self-efficacy, enactive attainment, knowledge, and outcome expectations [32]. Behavior change techniques are an important addition to the patient feedback processes described above, as they promote patient engagement and uptake of DHIs.

We aimed to further develop the AF DHI on the Corrie Health platform to create a mature product that reflects the needs of diverse patients, caregivers, and clinicians. Furthermore, we aimed to summarize our findings from the process and to propose an approach to HCD tailored for DHI development in an inclusive manner: the Virtual Inclusive Digital Health Intervention Design to Promote Health Equity (iDesign) framework. This framework can be applied to the development and optimization of other DHIs with relevant stakeholder input. Our goal is to help guide clinical investigators and health app developers to create engaging, equitable, and scalable digital health solutions.

## Methods

### Study Setting

To conduct this qualitative research study, we recruited an expert designer from the Johns Hopkins Center for Health Equity (author NM) who had completed in-person training in HCD methodology. The expert designer led the design process along with a clinician (author NI) who had experience in caring for patients with AF and had also completed online training in HCD methodology. We also recruited 4 individuals to help capture the meeting discussions in real time, including 2 clinicians, 1 undergraduate student, and 1 postgraduate student.

### Ethics Approval

The study was approved by the Johns Hopkins Institutional Review Board (IRB00230621).

### Patient Recruitment

We conducted purposeful sampling with the goal of having representation from multiple ethnic and racial backgrounds. While we were able to enroll White, Black, and Asian individuals, we were not successful in enrolling Hispanic or Native American individuals due to the very small number of eligible individuals in the source population. We recruited 8 patients with a diagnosis of AF and their caregivers or health partners (if the patient chose to include them) who were scheduled to see a cardiologist or electrophysiologist within 6 months of enrollment. All participants were recruited virtually via phone call, and consent was obtained by email. Design meetings were conducted on the Zoom platform. Virtual study procedures were chosen to allow for a safe study process in the setting of the COVID-19 pandemic. This also allowed us to increase access and engage eligible patients with a limited ability to attend in-person visits due to barriers such as work, household circumstances, and transportation [33].

Inclusion criteria were a diagnosis of AF, ownership of a smartphone (iPhone 5 or newer), residency in the United States, and proficiency in English.

### Clinician Recruitment

**Figure 1:**
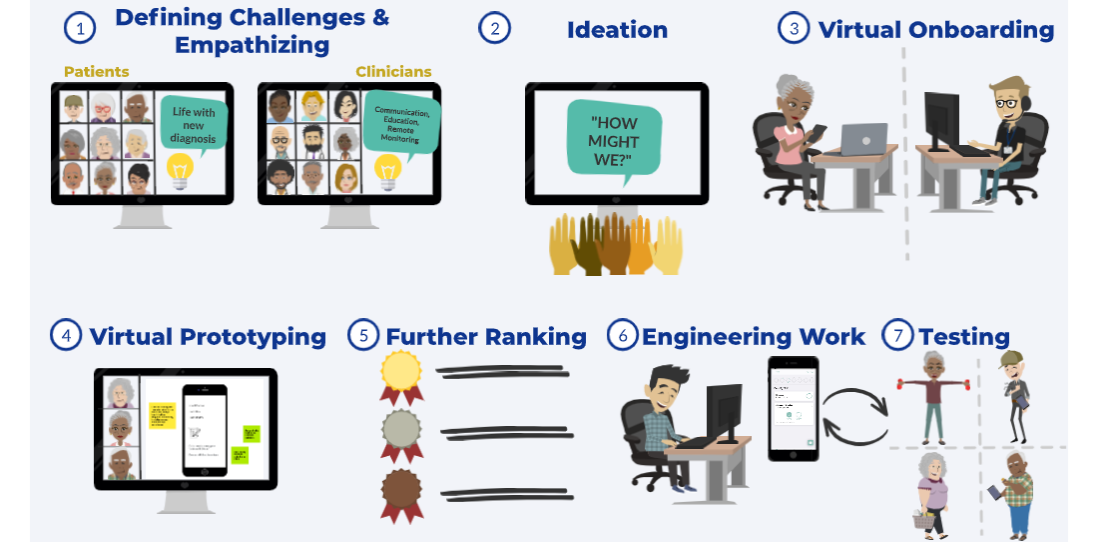
Steps of the Virtual Inclusive Digital Health Intervention Design to Promote Health Equity (iDesign) framework.

Clinicians were recruited using flyers and emails with information regarding the study. Eligible clinicians included cardiologists, electrophysiologists, pharmacists, and advanced care providers. All clinicians had more than 3 years of experience caring for patients with AF. All participants provided informed consent virtually via email, as discussed above. Methods for each step of the iDesign process are outlined in the following sections and are also summarized in Figure 1.

We strived to be as inclusive as possible throughout the process, within the limitations of the COVID-19 pandemic, by (1) including a health equity expert (who was also an expert in HCD) to co-lead the HCD sessions, (2) inviting patients, caregivers, and clinicians with different backgrounds and expertise to drive the development of the components of the AF DHI, (3) offering virtual study visits that allowed for the inclusion of individuals who would not be able to take time off from work or travel to in-person visits, (4) offering one-to-one technology onboarding to allow the participation of individuals with different levels of technology literacy, and (5) ensuring that everyone contributed throughout the meetings by providing enough time for participants to speak and to reflect at the end of the session on whether their ideas were correctly captured. While the study team carefully considered providing a loaner phone with a data plan to patients who did not own that technology or have internet access, we decided at the time that device recirculation may have posed an undue risk of COVID-19 transmission.

### Step 1: Defining Challenges and Empathizing

#### Patients

We conducted a virtual 1.5-hour meeting using audio-video conferencing on the Zoom platform with patients and caregivers or health partners. Discussions during the meeting were guided by the prespecified agenda. After introductions and an ice-breaking activity, the study leads explained to the participants that the goal of the session was for the study investigators to understand challenges patients experience while living with AF and to understand their journey after the diagnosis. Patients were also educated about the HCD approach and were reminded of the study goal to develop a DHI that would address the challenges of diverse patients with AF. Patients were divided into 2 groups to allow them to express their experiences with minimal time constraints. Participants were encouraged to think about either their personal experience as a patient or as a caregiver supporting a patient. We explained to participants that we would be using a journey map to capture their experiences following their AF diagnosis and their life with AF. We used the software Lucid (Lucid Software, Inc) to capture these experiences, which were projected on the shared Zoom screen. When the participants shared their experiences, we asked them to mark their timeline with notable milestones, which could be appointments, months, special holidays, or periods that marked how they were feeling. The study team used the following prompt during the breakout sessions to develop the journey map: “Let’s take a moment to think back to your first interaction with the healthcare team you had when diagnosed with AF. We want to hear your story as well as your partner’s story. Walk us through your journey after the diagnosis of AF.” We encouraged participants to focus on the following questions while answering the prompt: “What were your thoughts?” “How did you feel?” “What went well?” and “What were your challenges?”

We then shared the journey maps and the overall broad themes that emerged from the discussion with the whole group. The study participants were asked if their thoughts were adequately captured. Afterward, we thanked participants for their time and reminded them about the next steps, which included identifying top challenges that we would address together, followed by ideation, prototyping, and testing solutions.

Although we had identified broad themes during the sessions, we also conducted a qualitative analysis of a transcript of the audio recording of the session using the software ATLAS.ti (ATLAS.ti GmbH). First, each sentence was inductively coded via descriptive codes that were then clustered into broader codes sharing similar themes. These were further clustered into broad, emerging themes and formed into insight statements (these are discussed in the results section). Coding was conducted by the clinician-researcher (NI) and reviewed by an expert designer (NM) to ensure the dependability of the process.

#### Clinicians

We condensed step 1 (defining challenges) and step 2 (ideation) for the clinician meeting in order to accommodate the study participants’ schedules. We conducted a 1.5-hour meeting virtually via Zoom and recorded the session. Like the patient and caregiver meeting, the clinician meeting was guided by the prespecified agenda. After introductions and an ice-breaking activity, we asked the clinicians to share their responses to the following open-ended questions: (1) “What challenges do you face while caring for patients with AF?” and (2) “What are some patient strategies that increase their success in AF management?”

We summarized clinician responses using virtual whiteboarding and affinity diagraming. Affinity diagraming includes grouping together conceptually similar notes captured during discussion and identifying broad, emerging themes. Broad themes that were identified from the clinician sessions were then transformed into insight statements.

### Step 2: Ideating Solutions (Patients Only)

Following step 1, a qualitative analysis of the meeting script was used to identify broad themes, which were converted into insight statements, as outlined above. We then constructed “how might we” questions from the insight statements. “How might we” questions are core components of the HCD approach and are meant to promote creativity [34]. We conducted a 1.5-hour meeting via Zoom with the goal of brainstorming and ideating solutions for the top challenges patients and caregivers expressed during the first meeting. We asked participants if the list of “how might we” questions generated from the transformation of emerging themes reflected what they shared during the first meeting. Participants were given the freedom to add or modify any questions. After confirming the accuracy of the “how might we” question list, we asked everyone to vote on the top-4 priority questions they wanted to brainstorm about.

Following the guidance of our expert human-centered designer, we decided to deploy the existing technology after the ideation phase. This was done to avoid bias in the thought process and facilitate generation of creative ideas without exposing participants to the existing technology. Additionally, we felt it was necessary for participants to have an understanding of existing capabilities and overall app design prior to engaging in prototyping.

### Step 3: Individualized Virtual Onboarding (Patients Only)

We set up a Zoom meeting at a convenient time for each study participant to conduct individualized virtual onboarding to the minimally viable version of the Corrie app, which we named Corrie Afib. We followed a systematic approach for onboarding participants, which is described in Multimedia Appendix 1. The time required for onboarding each participant varied considerably, ranging from 15 minutes to 2 hours, based on the participant’s digital health literacy. Patients attended this session without caregivers.

### Step 4: Virtual Prototyping (Patients Only)

During the third 1.5-hour Zoom meeting, we first asked the study participants to identify the top solutions they wanted to design from the list of solutions that were generated during the second meeting (discussed in the results section). Study participants then voted using the Zoom chat box on the top solutions they wanted to prototype. The participants were split into 3 groups, and each group was given the task of prototyping the intervention. Virtual prototyping was conducted using Google Jamboard, which lets study designers capture patients’ design ideas in real time and allows them to confirm their accuracy by continuously asking questions.

### Step 5: Further Ranking

Additional ranking with study investigators and engineers was conducted, because the patients expressed their desire for other solutions to be incorporated in the DHI, although due to the time constraints of the virtual meetings, prototyping of only 3 solutions was feasible. Three study coinvestigator-clinicians and engineers met for discussion and ranked more than 30 patient ideas and more than 20 clinician ideas (Multimedia Appendices 2 and 3) that were not prototyped by patients; the ranking was based on feasibility, viability, and desirability and used a 3-point Likert scale [35]. Top-scoring solutions were prioritized for incorporation into the app, in addition to the ideas chosen by patients for prototyping. Our approach, like the HCD process, was flexible. When feasible, the ranking process can be combined with the ideation session. We then leveraged the Theory of Health Behavior Change as a conceptual model to guide further incorporation of the features in the design process [36,37]. The Theory of Health Behavior Change considers 3 major components driving individual behavior change: knowledge and beliefs, self-regulation skill and ability, and social facilitation (Multimedia Appendix 4) [36,37].

### Step 6: Engineering Work (Ongoing)

Once specific functionalities were prioritized, the engineering team began to integrate the selected features into the app.

### Step 7: Testing (Future)

The app functionalities informed by our iDesign process will be modified iteratively until no further major feedback is obtained from pilot clinical testing in a larger group of patients (N=100), who will be either Android or iOS users.

## Results

### Step 1: Defining Challenges and Empathizing

#### Patients

Patients (N=8) and their caregivers or health partners (N=3) joined the first group discussion virtually over the Zoom platform. Subsequent sessions were attended only by patients, without caregivers or health partners. The median age of the patients was 69 (IQR 58-78) years. There were 2 Black participants, 1 Asian participant, and 4 women; 4 patients had commercial insurance and 4 patients had Medicare insurance.

After conducting a qualitative analysis, we identified the following insight statements: (1) addressing risk factor modification and medication adherence is important; (2) guidance during AF episodes is important, because the episodes cause anxiety; (3) patients seek more information and education regarding AF, particularly regarding life after AF diagnosis; (4) patients seek education on medications and need help with medication adherence; (5) patients need to understand therapy options; and (6) patients need peer support. We then converted these insight statements into the following “how might we” questions (Textbox 1).

**List of “how might we” questions.**

**“How might we” questions (patients)**

1. “HMW [how might we] use the Afib [atrial fibrillation] app to guide me in making lifestyle changes?”

2. “HMW use the Afib app to better guide me when I have symptoms of Afib?”

3. “HMW use the Afib app for education after diagnosis of Afib?”

4. “HMW use the Afib app to manage my medications?”

5. “HMW use the Afib app to help me better understand different options of care when living with Afib?”

6. “HMW use the Afib app for community support?”

**“How might we” questions (clinicians)**

7. “HMW better educate patients using the Afib app?”

8. “HMW better communicate with patients using the Afib app?”

#### Clinicians

We recruited 8 clinicians (including 2 women), although 2 of the recruited clinicians were not available for the meeting due to a schedule conflict. As described above, we combined defining the problems (ie, empathizing) and ideating solutions into 1 meeting for the clinicians. We identified the following insight statements through affinity diagraming during the session: (1) patient education is inadequate; (2) addressing AF modifiable risk factors is a priority; and (3) remote AF management and communication are needed.

We then constructed “how might we” questions (Textbox 1) based on these insight statements. Notably, insight statements from patient and caregiver input were similar to the ones generated by clinicians.

### Step 2: Ideating Solutions

#### Patients

Patients selected the top 3 questions from Textbox 1 that they felt were most desirable to address. The top questions selected were questions 1, 2, and 5. The broad range of creative ideas brainstormed by the patients, numbering more than 30, are outlined in Multimedia Appendix 2.

#### Clinicians

The broad range of creative ideas brainstormed by clinicians, numbering more than 20, are outlined in Multimedia Appendix 3.

### Step 3. Individualized Virtual Onboarding (Patients Only)

After one-on-one onboarding sessions with patients, we identified several learning points to further streamline the process for future studies. First, prior to connecting with the patient via Zoom, the study team member should send an introductory email explaining to the patient what they need prior to onboarding, including (1) their Apple ID and password or Google Play ID and password, (2) a stable Wi-Fi connection, and (3) a smartphone or, ideally, another device (such as a laptop, iPad, or computer) where they can watch an explanation of the onboarding process by the study team member while downloading the app to their phones and completing onboarding. Second, there is a need for 2 versions of onboarding videos, one for individuals who are more comfortable using technology and the other for those who need more guidance, to streamline the process while still addressing the needs of diverse individuals. The video directed toward individuals who are comfortable with technology should be brief and should only show each feature and give one example of using that feature, while the version directed toward individuals with low digital literacy should review the details of how to open an email from the study team and download the app, retrieve the passwords needed for app installation, set up an automated login (if desired), and give more than one example of using each feature of the app. Third, the study team should be available for questions and technical support.

### Step 4: Virtual Prototyping (Patients Only)

At the end of the ideation meeting, we asked patients to vote on the top 3 ideas that they felt were most desirable to prototype. Table 1 shows the voting results.

Participants were then split into 3 groups, and the groups were given the task of prototyping each feature. Models of working prototypes were created with patient input for (1) providing information about AF triggers (Multimedia Appendix 5), (2) symptom and rhythm correlation for alerting the clinician team and family while experiencing AF (Multimedia Appendix 6), and (3) improved medication tracking (Multimedia Appendix 7).

**Table 1:** Patient choices on what to prototype and forming “what to prototype” statements.

What to prototype?	Patient choices from ideation session
AF^a^ triggers	Include information about AF triggers.
How to know you are having AF when you are or are not having symptoms; how the app can guide you during an episode	Have a way to know if you are having AF when you do not recognize symptoms, such as by providing heart rate and blood pressure. Have a feature to learn if a fast heart rate is from AF or something else. Provide the ability to connect with clinicians, friends, or family via the app when you have symptoms, to see if they can help with the decision on what to do next. Quicker connection to people, especially during night hours, to help make decisions about next steps.
Improved medication tracking	Include information on side effects of medications and what different options are available. Include information on the effects of medications on lifestyle. Indicate what medication and dose to take, and provide a reminder to take the medication at the correct time. Include pictures of the different medications to help recognize them.

^a^AF: atrial fibrilation.

### Step 5: Further Ranking

A second round of ranking identified patient education, peer support, and clinician engagement in remote AF management as viable, feasible, and desirable functionalities to incorporate within the app. New or modified functionalities chosen to be incorporated were aligned with the components of the Theory of Health Behavior Change (Multimedia Appendix 4).

### Steps 6 and 7: Engineering Work and Testing

Engineering and development are in progress to expand the feature scope of the Corrie Afib app and the clinician dashboard based on HCD. Features include an expanded library for tailored AF education, risk factor tracking, rhythm monitoring for potential triggers, symptom and rhythm correlation for better therapeutic guidance, and community support (Multimedia Appendix 4).

Timeline for each step of the design process is included in Multimedia Appendix 8. Even after completion, this iterative process will continue with feedback obtained from patients enrolled in a pilot study of 100 participants.

## Discussion

### Principal Findings and Literature Review

In this exploratory co-design project, patients, their caregivers, and clinicians identified salient challenges in AF care, including inadequate education and guidance on AF management topics, such as lifestyle changes and guideline-directed medical therapy, inadequate resources to guide decision-making during an AF episode, and poorly structured patient-clinician communication during AF episodes. Similar qualitative and co-design experiences have not yet been described for AF management, but the challenges identified by our study participants overlap with known gaps in AF care outlined in the literature. In line with our participants’ identification of inadequate education and guidance on AF management, there is evidence that a significant proportion of individuals with AF have inadequate disease-specific health literacy [26]. Furthermore, anticoagulation therapy, a core component of AF management to prevent stroke, is only implemented in less than half of individuals who are at high risk for stroke [25]. Significant disparities in stroke prevention therapy also exist, as women and Black individuals receive anticoagulation therapy less often than men and White individuals [24,27]. In addition, Black and low-income individuals are less likely to receive rhythm control therapy, including catheter ablation [23]. Educating and empowering patients regarding these core principles of AF management, as suggested by the patients and clinicians, has the potential to improve implementation of guideline-directed therapies. We co-designed and coprototyped solutions together with patients, caregivers, and clinicians and identified top features that will inform further development of AF DHIs to address the identified challenges. Further feasibility of our AF DHI will be assessed in a pilot study and, subsequently, in a definitive clinical study to assess its efficacy in improving quality of life and cardiovascular outcomes.

To date, HCD concepts have been applied in the design of DHIs for management of diseases including diabetes, heart failure, and metabolic syndrome [10,38,39]. A company specializing in HCD was asked to design an app for Ascensia Diabetes Care Holdings AG with the goal of educating and monitoring patients with diabetes; however, a detailed description of the methods is not available [34]. Joshi et al [38] engaged 10 dietetic interns to define problems, ideate, and prototype a mobile app for patients with metabolic syndrome. While clinicians were engaged in the design process, patient-level input, which is critical to meeting the needs of end users, was missing. Zachari et al [39] designed a diabetes self-management app using participatory design, in which understanding patient challenges through empathy was at the center of the design process; however, clinician involvement was missing, as was the case for the design of a heart failure digital tool [10]. Ahmed et al [11] described an investigation targeting the need for development of an integrated care platform for individuals with neurodegenerative disease, incorporating feedback from patients, clinicians, and other stakeholders with a mixed methods approach; however, the ideation component of HCD was not reported in that study. Given the limited literature on using comprehensive methodologies to apply HCD principles specifically to DHI design, we used this paper to summarize our experience in developing an AF DHI and to outline our design framework, iDesign, as an approach to HCD (Figure 1). The iDesign framework is based on core HCD principles and can be used as a template for investigators planning to develop or optimize DHIs. Throughout the co-design process, we have made a number of findings that will help promote an inclusive design process and streamline the iDesign framework for future innovative projects (Multimedia Appendix 9). Future study teams may build on or modify individual components of the iDesign process as deemed appropriate for particular scenarios. The iDesign framework may serve as an overall guide for the process of DHI development for disease management.

### Limitations

First, while our study engaged a diverse group of patients, caregivers, and clinicians in the co-design process, our sample size was modest, and we may not have captured insights from participants who do not speak English or have uncommon backgrounds and experiences. However, our study included participants from various backgrounds, and both the patients and clinicians shared similar challenges and experiences, suggesting that we attained inductive thematic saturation. Furthermore, clinicians with years of experience caring for patients with a rich array of backgrounds were engaged as stakeholders, which may have counterbalanced this limitation. In a subsequent stage of testing the AF DHI, we will recruit a larger sample size with broader representation to solicit further feedback. Second, while the virtual co-design approach facilitated inclusiveness by overcoming barriers such as transportation or work schedules, a virtual environment may not be ideal for participants who have limited technology skills or do not have access to technology or the internet. By engaging patients with various levels of comfort with technology in our design process, we learned the significance of the onboarding process, in which skills can be taught to help overcome the technology-literacy challenge. To overcome a lack of access to technology and barriers to internet access, we plan to work with local communities, as well as policy makers, insurance agencies, and other stakeholders to facilitate both design and further implementation of the DHI.

### Conclusions

We identified key recommendations from patients, caregivers, and clinicians with diverse backgrounds that informed the development of a DHI for AF management on the Corrie Health digital platform. We have extended our design process experience and formulated a structured inclusive framework, iDesign, which stems from components of HCD, for informing the development and optimization of DHIs. This framework adds to the body of knowledge on approaches to HCD, is flexible, and is transferable to the development and adaptation of DHIs.

## Appendix

Multimedia Appendix 1 Steps of the onboarding process to the Corrie Afib application.

Multimedia Appendix 2 Responses to “How Might We” questions during the patient ideation session.

Multimedia Appendix 3 Responses to “How Might We” questions during the clinician ideation session.

Multimedia Appendix 4 Theoretical basis of behavior change strategies in Corrie Afib.

Multimedia Appendix 5 "What triggers Atrial fibrillation" feature prototype design by patient input.

Multimedia Appendix 6 “Way to know you are having Atrial fibrillation when you are or aren’t having symptoms and how the application can guide you during an episode” feature design prototype.

Multimedia Appendix 7 “Improve medication adherence” feature design prototype.

Multimedia Appendix 8 Time Required for each step of the design process.

Multimedia Appendix 9 Guidance on using Virtual (i)nclusive Digital Health Intervention Design to Promote Health Equity (iDesign) Methodology.

## Abbreviations

AF: atrial fibrillation
DHI: digital health intervention
HCD: human-centered design
iDesign: Virtual Inclusive Digital Health Intervention Design to Promote Health Equity
