# Supplementary material for: The Virtual Inclusive Digital Health Intervention Design to Promote Health Equity (iDesign) Framework for Atrial Fibrillation: Co-design and Development Study
Source: JMIR Hum Factors. 2022 Oct 31;9(4):e38048. doi: 10.2196/38048 (PMC9664334; doi:10.2196/38048)
Supplement: Multimedia Appendix 1 [file humanfactors_v9i4e38048_app1.docx]

**Multimedia Appendix 1.** Steps of the onboarding process to the Corrie Afib application

| **Steps** | **Step Details** |
| --- | --- |
| 1. Welcome | We welcomed participants to the onboarding  session. |
| 2. Download & Corrie Account Set Up | We sent an email to participant with a link to download the Corrie Afib app from the app store at the beginning of the meeting. We walked them through the process of downloading the app, setting up an account and Face or Touch ID if  desired, and setting up the Corrie Afib app. |
| 3. Configuration | We then shared with participants that they had options to (1) enable permissions on their phone to share health data with Corrie and (2) enter  profile information if desired. |
| 4. Corrie Functionality Overview | We then walked patients through different functionalities of Corrie by sharing the screen of a  study team member’s phone via Zoom®. |
| Afib = Atrial fibrillation, app= Application, ID = Identification | |
