# Supplementary material for: The Virtual Inclusive Digital Health Intervention Design to Promote Health Equity (iDesign) Framework for Atrial Fibrillation: Co-design and Development Study
Source: JMIR Hum Factors. 2022 Oct 31;9(4):e38048. doi: 10.2196/38048 (PMC9664334; doi:10.2196/38048)
Supplement: Multimedia Appendix 2 [file humanfactors_v9i4e38048_app2.docx]

**Multimedia Appendix 2.** Responses to “How Might We” questions during the patient ideation session.

| **HMW use the AF app to guide me in making lifestyle changes?** | **HMW use the AF app to better guide me when I have symptoms of Afib?** | **HMW use the AF app to help me better understand different options of care when living with**  **AF?** |
| --- | --- | --- |
| Have a list of healthy and unhealthy foods | Have a feature to learn how fast heart is beating and to know if it’s an emergency | App should indicate what medication and dose to take, and provide a reminder to take at the correct time |
| Include information on portions, e.g.  how many cups of coffee/day is safe, how much salt etc. | Have a feature to notify you when you have irregular heartbeat | Include pictures of the different  medications to help recognize them |
| Include information regarding appropriate level of exercise (too little, too much), weight loss (what is too much), healthy weight | Have a feature to learn if fast heart rate is from AF or something else | Include information on side effects of medications and what different options are available |
| Include information on supplements with impact on AF and effects of blood thinners | Have feature to provide guidance on ways to reduce symptoms and elevated heart rate and guidance on what to do when you are having episodes. e.g. breathing exercises | Include information on what are the next steps if medications are not helping, e.g. procedure so patient knows what to do next |
| Have ability to track physical activity, weight, and sleep | Have a feature to allow tracking of how long each episode lasts over time so you can compare and calculate how many episodes you have had. Be able to take notes on what you are doing during that time, to try to find correlations | App should include up to date information about currently available procedures and risks to help make decision, especially for older adults |
| Have ability to access a clinician for individualized lifestyle changes and personalized planning | Have a feature that lets you create an alarm if you know you have symptoms from AF so you can get help immediately | Have websites to direct people to, to support people with AF |
| Include information about AF triggers | Have a way to know if you have AF when you aren’t recognizing symptoms. e.g. by giving you heart rate and blood pressure | Have app contents in different languages so people all over world can use the app |
| Include positive examples of people who live with AF and make healthy lifestyle changes as directed for care | Have ability to connect with clinicians, friends/family via the app when you have symptoms to see if they can help with decision on what to do next. Quicker connection to people, especially during night hours, to help you make decision about next steps | Have more information on different levels of AF severity and whether an individual will inevitably have more severe AF |
| Include vocabulary on AF terms | Have feature that guides you at what point do you go to the hospital/emergency room. Would want to know, if based on duration, e.g. 200 bpm for 5 minutes, is it okay to stay home or should you go to the emergency room? | Have comprehensive information to allow for better understanding of what a patient with AF is dealing with and what to expect, so they can learn what is next  early on. Have education on how to prepare to prevent severe AF |
| Have feature to plug in question on  diet/exercise and be able to get an answer | -- | Include information about hereditary component of AF |
| Include a resource, i.e. a place to go  to ask questions | -- | Include information on the effect  of medications on lifestyle |
| Have the ability to track hydration (alarm to make sure you are staying  on track with hydration) | -- | -- |
| AF = Atrial Fibrillation, Bpm = beats per minutes, HMW = How Might We | | |
