# Supplementary material for: The Virtual Inclusive Digital Health Intervention Design to Promote Health Equity (iDesign) Framework for Atrial Fibrillation: Co-design and Development Study
Source: JMIR Hum Factors. 2022 Oct 31;9(4):e38048. doi: 10.2196/38048 (PMC9664334; doi:10.2196/38048)
Supplement: Multimedia Appendix 3 [file humanfactors_v9i4e38048_app3.docx]

**Multimedia Appendix 3.** Responses to “How Might We” questions during the clinician ideation session.

| **HMW better educate patients and promote risk**  **factor modification using the AF app?** | **HMW better communicate with patients using the AF app?** |
| --- | --- |
| Include education on the importance of taking medications they are prescribed | Apps can have a feature to help patients decide when to involve their cardiologist. A 24 hours response time to patients would be reasonable for non-urgent  questions |
| Include education on stroke risk, especially for patients with asymptomatic AF | Incorporate a tool to determine whether patients are in AF when not feeling well |
| Provide different levels of information for patients with different levels of education | Include connection to an advanced practice provider via the app |
| Educate patients on importance of early decision of rhythm control | Provide positive reinforcement from the app for patients who track their vital signs |
| Create educational content (e.g. 5 min video prior to clinic visit) | A triage/communication service with a stratified action plan led by advanced practice providers can be built within the app |
| Deliver AF education at the time of the discharge | Feature to allow sending a screenshot of a single lead ECG output to an advanced practice provider via the app can help reduce physician load and reassure patients |
| Link in professional society resources into the app | Stratified action plans for a confirmed AF episode |
| Consider linking in prescribing information for each medication | Feature that would facilitate communication with patients |
| Incorporate information on drug-drug interaction | Create space in the app to store single lead ECG recordings to determine AF burden |
| Incorporate shared decision-making tools |  |
| Empower patients by tracking vitals, step count, weight |  |
| Incorporate a checklist of the modifiable AF risk factors |  |
| AF = Atrial Fibrillation, ECG = Electrocardiogram | |
