# Supplementary material for: The Virtual Inclusive Digital Health Intervention Design to Promote Health Equity (iDesign) Framework for Atrial Fibrillation: Co-design and Development Study
Source: JMIR Hum Factors. 2022 Oct 31;9(4):e38048. doi: 10.2196/38048 (PMC9664334; doi:10.2196/38048)
Supplement: Multimedia Appendix 4 [file humanfactors_v9i4e38048_app4.docx]

**Multimedia Appendix 4.** Theoretical basis of behavior change strategies in Corrie Afib

| Theory of Health Behavior Change | Ideas selected from patient and clinician feedback (Multimedia Appendix 2 and 3, including features prototyped by patients) | Feature in Corrie Afib |
| --- | --- | --- |
| - Knowledge and Beliefs - Condition Specific Knowledge - Personal Perceptions - Self -efficacy - Outcome expectancy - Goal congruence | - Include education on stroke risk, especially for patients with asymptomatic AF - Include information about AF triggers - Educate patients on importance of early decision on rhythm control - Create educational content (e.g., 5 min video prior to clinic visit) - Incorporate a checklist of the modifiable AF risk factors - Include education on the importance of taking prescribed medications - Include information regarding appropriate level of exercise (too little, too much), weight loss (what is too much), healthy weight - Link professional society resources into the app - Provide different levels of information for patients with different levels of education - Include information on side effects of medications and what different options are available - Have comprehensive information to allow for better understanding of what a patient with AF is dealing with and what to expect, so they can learn what is next - Have education on how to prevent severe AF | - Educational articles, videos and key resources for AF risk factors/triggers, benefits of risk factor control have been created for AF patients. Resources provide a range of informational depth to accommodate readers with different levels of literacy, including links to professional society material. - Information about medication side effects exists in Corrie App |
| - Self-regulation Skill and Ability - Goal setting - Self-monitoring & reflective thinking - Decision making - Planning and plan enactment - Self-evaluation - Management of emotional response | - Have a way to know if you have AF when you aren’t recognizing symptoms - Have a feature to learn if a fast heart rate is from AF or something else - Have ability to connect with clinicians, friends/family via the app when you have symptoms to see if they can help with decision on what to do next. Quicker connection to people, especially during night hours, to help decide next steps - Have ability to access a clinician for individualized lifestyle changes and personalized planning - Create space in the app to store single lead ECG recordings to determine AF burden - Incorporate a tool to determine whether patients are in AF when not feeling well - Feature that would facilitate communication with patients - Have ability to track physical activity, weight, and sleep - Empower patients by tracking vitals, step count, weight | - Rhythm check feature, rhythm, potential AF trigger and symptom correlation feature along with summary of results being developed to allow AF episode recognition and guide next steps in management - Feature to allow clinical team, family, and emergency contact information is built into app for easy connection - Ability to track physical activity, step count and weight exists in the App - Ability to integrate wearable collected data on sleep and sleep apnea therapy adherence - Ability to track medications and their side effects already exists in the App |
| - Social Facilitation - Influence - Support (emotional, instrumental, information) | - Include a resource, i.e., a place to go to ask questions | - Feature to allow access to a peer support Facebook page is integrated to help build community by providing emotional and informational support |
| AF = Atrial Fibrillation | | |
