# Supplementary material for: The Virtual Inclusive Digital Health Intervention Design to Promote Health Equity (iDesign) Framework for Atrial Fibrillation: Co-design and Development Study
Source: JMIR Hum Factors. 2022 Oct 31;9(4):e38048. doi: 10.2196/38048 (PMC9664334; doi:10.2196/38048)

**Multimedia Appendix 5.** “What triggers Atrial fibrillation" feature prototype design by patient input.


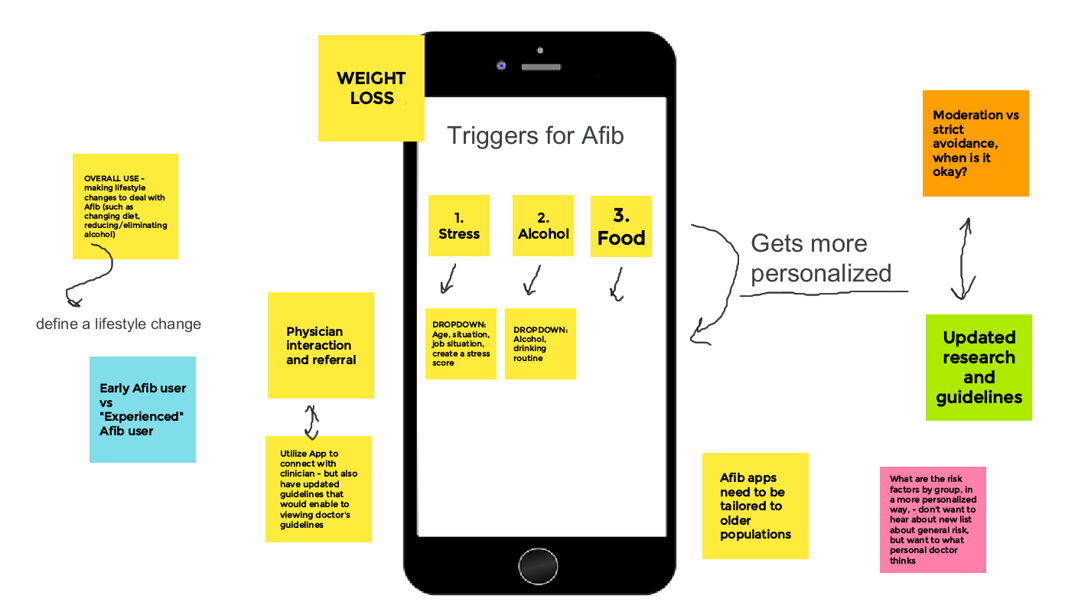

Supplement: Multimedia Appendix 5 [file humanfactors_v9i4e38048_app5.docx]
