# Supplementary material for: The Virtual Inclusive Digital Health Intervention Design to Promote Health Equity (iDesign) Framework for Atrial Fibrillation: Co-design and Development Study
Source: JMIR Hum Factors. 2022 Oct 31;9(4):e38048. doi: 10.2196/38048 (PMC9664334; doi:10.2196/38048)
Supplement: Multimedia Appendix 6 [file humanfactors_v9i4e38048_app6.docx]

**Multimedia Appendix 6.** “Way to know you are having Atrial fibrillation when you are or aren’t having symptoms and how the application can guide you during an episode” feature design prototype.

**
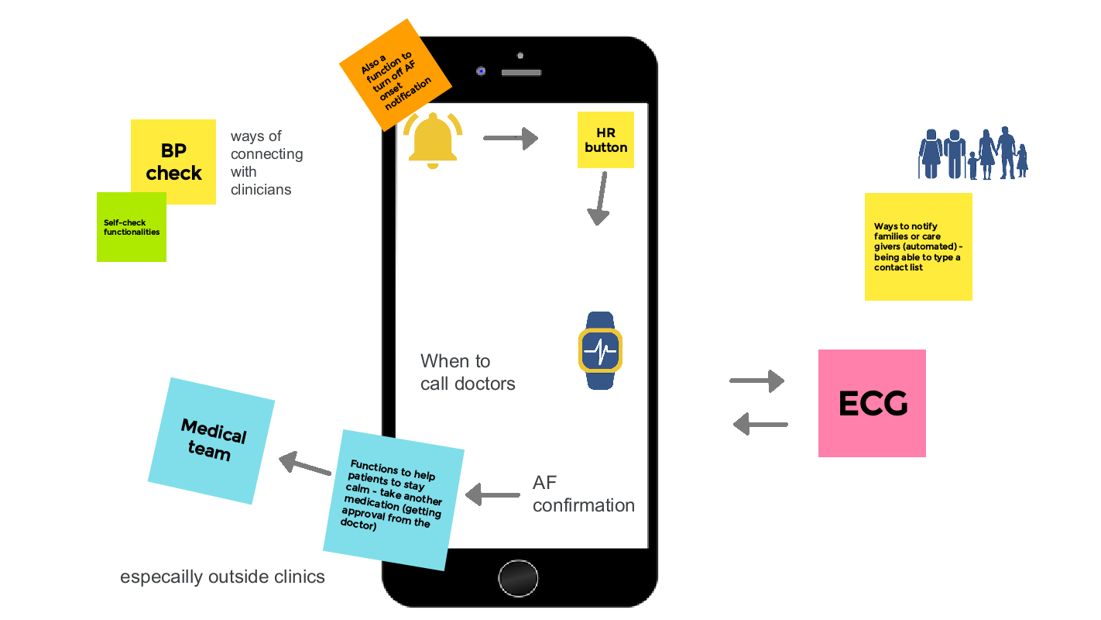
**
