# Supplementary material for: The Virtual Inclusive Digital Health Intervention Design to Promote Health Equity (iDesign) Framework for Atrial Fibrillation: Co-design and Development Study
Source: JMIR Hum Factors. 2022 Oct 31;9(4):e38048. doi: 10.2196/38048 (PMC9664334; doi:10.2196/38048)

# Multimedia Appendix 7. “Improve medication adherence” prototype. Prototypes were captured by study team members using virtual whiteboarding in real-time in response to patient input. While exact statements in the “sticky notes” are not visible we show them for the purpose of demonstrating the process.

#
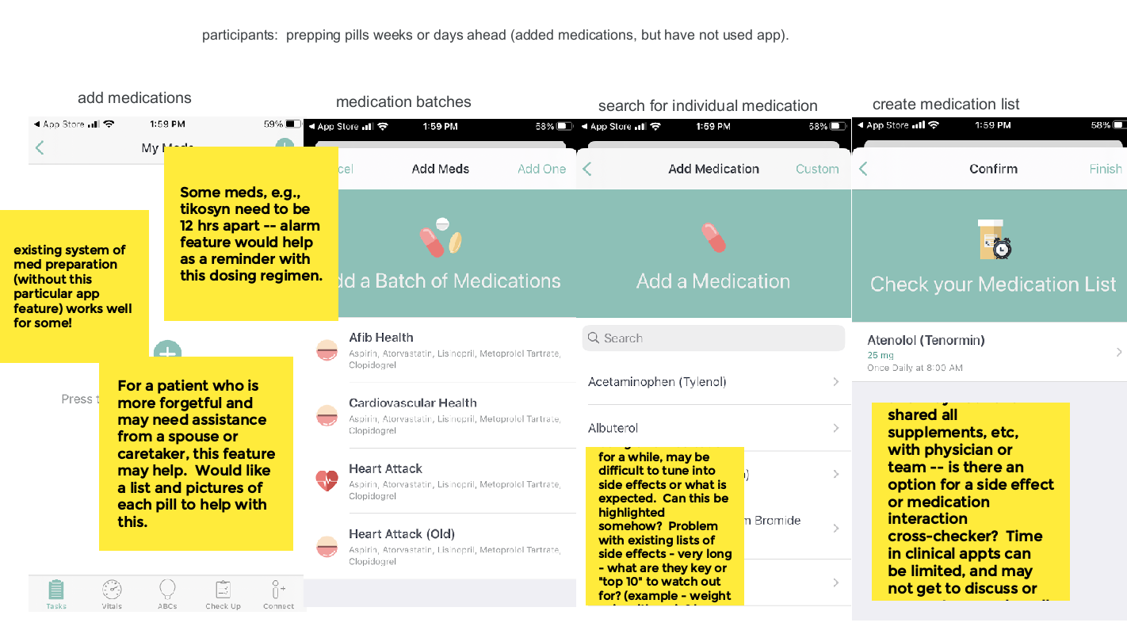

Supplement: Multimedia Appendix 7 [file humanfactors_v9i4e38048_app7.docx]
