# Supplementary material for: The Virtual Inclusive Digital Health Intervention Design to Promote Health Equity (iDesign) Framework for Atrial Fibrillation: Co-design and Development Study
Source: JMIR Hum Factors. 2022 Oct 31;9(4):e38048. doi: 10.2196/38048 (PMC9664334; doi:10.2196/38048)
Supplement: Multimedia Appendix 8 [file humanfactors_v9i4e38048_app8.docx]

**Multimedia Appendix 8.** Time Required for each step of the design process

| Step | Patients | Clinicians |
| --- | --- | --- |
| 1-2 | Session Planning – One WeekTotal session duration - 3 hoursQualitative data analysis – 2 weeks | Session Planning – One WeekSession duration - 1.5 hoursQualitative data analysis – 2 weeks |
| 3 | 48 hours | - |
| 4 | Session Planning – One WeekSession Duration 1.5 hours | - |
| 5 | Ranking – 2 weeks | Ranking – 2 weeks |
| 6 | Ongoing | Ongoing |
| 7 | Pilot Study – planned over 1 year | Pilot Study – planned over 1 year |
