# Supplementary material for: The Virtual Inclusive Digital Health Intervention Design to Promote Health Equity (iDesign) Framework for Atrial Fibrillation: Co-design and Development Study
Source: JMIR Hum Factors. 2022 Oct 31;9(4):e38048. doi: 10.2196/38048 (PMC9664334; doi:10.2196/38048)
Supplement: Multimedia Appendix 9 [file humanfactors_v9i4e38048_app9.docx]

# Multimedia Appendix 9. Guidance on using Virtual (i)nclusive Digital Health Intervention Design to Promote Health Equity (iDesign) Methodology

*Key study planning considerations*

1. Create a team of designers, consisting of an expert designer and a clinician. Ideally the clinician should also receive design training. Combining the skills of an expert designer with the disease specific knowledge of a clinician is uniquely complementary and allows planning of the study procedures and meeting guides in a way that can yield more creative results that are applicable to study goals.
2. Health condition management is complex and involving multiple stakeholders in addition to the patient, who is central to the process can increase acceptability of the intervention. Engage indirect intervention users, including patient caregivers/health partners (if available), clinicians or other stakeholders as study participants to ensure a more holistic approach to DHI development or optimization.
3. Conduct purposeful sampling for group meetings to ensure inclusion of individuals with diverse sociodemographic backgrounds so that the end product can be scaled.
4. Conduct separate patient-caregiver and clinician design meetings to reduce bias from patients and caregivers/health partners with clinician views and vice versa and consider recording all meetings.
5. If applicable, consider loaning devices and/or providing internet access to participants for the duration of the study to ensure inclusion of individuals with lower socioeconomic backgrounds or technology literacy.
6. If applicable, tailor the onboarding experience to participants based on their technology literacy level.
7. Conduct virtual design meetings instead of in-person sessions to promote equity and ensure that participants are not being excluded from participation due to limited access to transportation, living out of state, or inflexible work hours.

STUDY PROCUDURES/STEPS

*Step 1. Defining Challenges & Empathizing*

Empathy generation is a core component of the HCD approach which focuses on developing deep understanding of individuals who will be the beneficiaries of the DHI.^1^ While empathy generation and understanding user experience can be achieved by different methodologies including in-depth interviews, group discussions, direct observation and analogous inspiration, we have found group discussions to be an effective way of identifying user experiences and needs for health conditions.

Step1 Guide:

- During the first meeting, consider capturing participant experiences in real time using whiteboarding (journey maps, sticky notes, text boxes, etc.). To ensure that the study team captures what the participant is sharing correctly, consider to screen share the whiteboard so participants can correct study team members if discrepancies are noted.
- An experienced designer can then identify broad emerging themes and group different study participant challenges together to create themes. This process is called affinity diagramming. In addition, the study team can^1^ conduct qualitative analysis of the recorded script of the meeting and identify themes via inductive coding.
- After the emerging themes are identified, the designer and clinical lead can transform them into short statements reflecting core insights.^1^
- If study investigators combine Step 1 and 2, similar to our approach for clinician sessions, affinity diagramming conducted live during the session is an optimal approach.
- The study leads can then transform the themes/core insight statements into “How Might We” questions. Transforming insight statements into “How Might We” questions requires experience and when done correctly they are excellent drivers for creativity.^1^

*Step 2. Ideation*

Step 2 Guide:

- Consider asking meeting participants to rank “How Might We” questions they consider most important to address. This can be performed by asking participants to write top three questions in the chat box or any other way study designers find feasible.
- The goal of this step, brainstorming/ideation, is to generate as many ideas as possible. The HCD field guide recommends that designers remind participants of the following factors that promote a successful brainstorming session: “deferring judgement, encouraging wild ideas, building on the ideas of others, staying focused on the topic, one conversation at a time, being visual, and going for quantity of ideas”.^1^
- Similar to step 1, we recommend that the study team captures participant ideas in a real-time.

At the end of the meeting, designers should consider pausing to ask study participants whether their ideas were captured accurately.

- Subsequently, participants can be asked to review all ideas generated and captured during the discussion and to identify their top three most desirable, viable, and feasible ideas that they would like to prototype.
- Study investigators might have a minimal viable product of the DHI or even a more developed product which they desire to advance using the iDESIGN process. In this case, Step 3 or onboarding to technology is needed to structure the design process around existing technology platforms. We do not recommend including technology onboarding at an earlier stage in the design process as this might introduce bias and limit the creativity of the brainstorming session.

*Step 3. Individualized Virtual Onboarding (Only applicable if platform already exists on which DHI is being built)*

Step 3 Guide:

- Consider conducting virtual 1:1 meetings with study participants to onboard them with the technology.
- The study team should be equipped with technology to ideally share their screen with the study participants and walk them through the DHI installation process, explain different functionalities in the DHI (if applicable) and answer any questions participants might have.
- The study team should ensure participants are comfortable with using the test version of the DHI before concluding the session.
- The study team should encourage participants to use the DHI in everyday settings and ask individuals to provide verbal or written feedback or both.
- Study team might also consider pre-recording two versions of the onboarding videos for individuals with different level of technology literacy. If onboarding is decided to be conducted asynchronously, study team can make support available via phone and/or email if further assistance is needed.

Step 4. *Virtual prototyping*

Step 4 Guide:

- - When designing a DHI with study participants who may have limited technology use literacy to allow them to work in the online collaborative platform to create prototypes, capturing participants’ prototype ideas in real time by study team members using platforms such as Google Jamboard® is an effective alternative. Real time whiteboarding allows designers to continuously check with participants whether their ideas are adequately captured.
  - When designing with individuals with great technology skills only, study participants can be provided with instructions on how to use virtual whiteboarding and collaborative platforms prior to the session so that they can perform the prototyping themselves live during the session.

*Step 5. Further Ranking*

Step 5 Guide:

Consider conducting further ranking of the ideas generated during the brainstorming meeting with study investigators, and engineers to identify other priority solutions that were not in the top three ideas selected by meeting participants during the STEP 2. We recommend the ranking methodology described by Aifah et al. ^2^ In this methodology, feasibility, viability and desirability (core principles of innovation) are each ranked on a 3- point Likert scale (ranging from not feasible, viable or desirable to most feasible, viable or desirable). Depending on the number of members who participate, a ranking cutoff, a score above which would indicate the prototype will be developed by engineers, should be established. Consider leveraging behavioral change theory and align selected new features to its components so that the final product is geared towards behavior change and can help build lasting healthy habits.

*STEP 6. Engineering work*

Step 6 Guide:

Depending on the difficulty level of the suggested DHI features, non-functional mock ups or fully functional features can be developed.

*STEP 7. Testing*

Step 7 Guide:

- - Consider inviting patients, their caregivers, clinicians and other stakeholders to use novel DHI functionalities in everyday life and provide feedback via 1:1 discussions, group meetings regarding the DHI functionalities.
  - Consider further DHI modification based on feedback. Iteration should continue until users report no major feedback.
  - Lastly, pilot testing of the DHI with a larger group of diverse patients, caregivers and clinicians should be performed to establish broad feasibility and usability of the DHI.

1. The Field Guide to Human-Centered Design By IDEO.org. Last accessed 2.20.2022. 2015.
2. Aifah A, Okeke NL, Rentrope CR, et al. Use of a human-centered design approach to adapt a nurse-led cardiovascular disease prevention intervention in HIV clinics. Prog Cardiovasc Dis. 2020;63(2):92-100.
